# Understanding Health-Related Quality of Life in Kidney Transplant Recipients: The Role of Symptom Experience and Illness Perceptions

**DOI:** 10.3389/ti.2023.10837

**Published:** 2023-04-13

**Authors:** Yiman Wang, Paul Van Der Boog, Marc H. Hemmelder, Friedo W. Dekker, Aiko De Vries, Yvette Meuleman

**Affiliations:** ^1^ Department of Clinical Epidemiology, Leiden University Medical Center (LUMC), Leiden, Netherlands; ^2^ Division of Nephrology, Department of Internal Medicine, Leiden University Medical Center (LUMC), Leiden, Netherlands; ^3^ Department of Nephrology, Maastricht University Medical Centre, Maastricht, Netherlands

**Keywords:** kidney transplantation, adult, illness perceptions, health related quality of life, symptom experience

## Abstract

The purpose of our article is to investigate the impact of symptom experience on health related quality of life (HRQOL) in kidney transplant recipients (KTRs) and whether illness perceptions mediated this impact. Symptom experience, illness perceptions, and HRQOL were measured at transplantation and 6 weeks after transplantation in KTRs in an ongoing Dutch cohort study. Multivariable linear regression models were used for the analysis. 90 KTRs were analyzed. Fatigue and lack of energy were the most prevalent and burdensome symptoms at transplantation. Mental HRQOL at 6 weeks after transplantation was comparable to that of the general Dutch population (mean [standard deviation, SD]: 49.9 [10.7]) versus 50.2 [9.2]), while physical HRQOL was significantly lower (38.9 [9.1] versus 50.6 [9.2]). Experiencing more symptoms was associated with lower physical and mental HRQOL, and the corresponding HRQOL reduced by −0.15 (95%CI, −0.31; 0.02) and −0.23 (95%CI, −0.42; −0.04) with each additional symptom. The identified mediation effect suggests that worse symptom experiences could cause more unhelpful illness perceptions and consequently lead to lower HRQOL. Illness perceptions may explain the negative impact of symptom experience on HRQOL. Future studies at later stages after kidney transplantation are needed to further explore the mediation effect of illness perceptions and guide clinical practice to improve HRQOL.

## Graphical Abstract

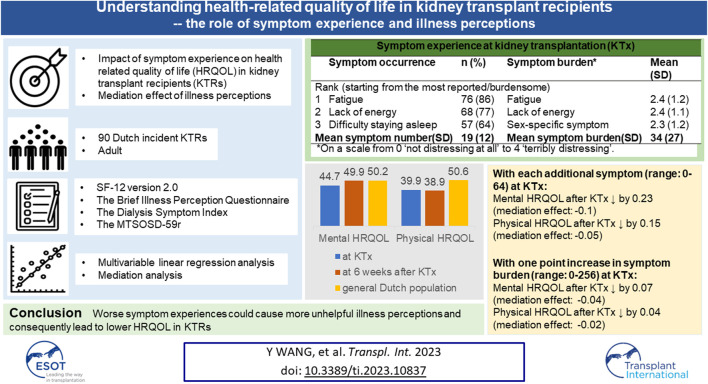

## Introduction

In patients with kidney failure, previous studies have shown the benefits of kidney transplantation regarding survival and health-related quality of life (HRQOL) compared to dialysis (1, 2). However, HRQOL after kidney transplantation is lower than that of the general population and healthy controls (1), which suggests room for further improvement. Therefore, it is of clinical interest to explore the risk factors for suboptimal post-transplant HRQOL and identify interventional targets for better health outcomes after kidney transplantation.

One potential risk factor for decreased HRQOL in kidney transplant recipients (KTRs) is the symptom experience, which comprises symptom occurrence and symptom burden. KTRs can experience a large number of symptoms and a high symptom burden due to their primary kidney disease (PKD) and the immunosuppresive treatment after kidney transplantation (3, 4). In patients with advanced chronic kidney disease (CKD) not receiving renal replacement therapy or on dialysis patients, existing evidence suggests an impact of the number of symptoms on HRQOL (5, 6). Previous studies in patients with other chronic conditions support these results and also found an association between high symptom burden and poor HRQOL (7, 8). Following Leventhal’s Common-Sense Model (CSM) of self-regulation, we hypothesize that the following mechanism could explain this association between symptom experience and HRQOL: symptoms are perceived as a health threat by patients, who then form cognitive and emotional illness beliefs and expectations about these health threats; these so-called “illness perceptions” shape patient’s behavioral and cognitive adjustment to managing their illness (i.e., coping strategy such as adherence to treatment and seeking support) which consequently contribute to health outcomes (Supplementary Figure S1) (9–11). Presumably, this could mean that the impact of symptom experience on HRQOL is mediated *via* illness perceptions. Previous research has indeed revealed associations between illness perceptions and various health outcomes (e.g., decline in kidney function and HRQOL) in patients with advanced CKD not receiving renal replacement therapy, dialysis patients and KTRs(12–16). However, to our knowledge, the mediation effect of illness perceptions between symptom experience and HRQOL has not yet been studied in CKD populations (including KTRs).

Therefore, our study explored the effect of symptom experience (i.e., symptom occurrence and burden) at transplantation on HRQOL 6 weeks after transplantation in Dutch incident KTRs (i.e., recently transplanted KTRs in relation to the study) and analyzed whether illness perceptions mediated this effect. Past research has shown that unhelpful illness perceptions are modifiable (17, 18), and hence, it is of clinical interest to understand whether illness perceptions can be a potential interventional target to alleviate the impact of symptom experience on HRQOL, especially in cases where effective treatments for symptoms are lacking.

## Patients and Methods

The STrengthening the Reporting of OBservational studies in Epidemiology (STROBE) guideline was used to guide the reporting of this study (19).

### Study Design and Participants

The Patient-reported OutcomeS In kidney Transplant recipients: Input of Valuable Endpoints (POSITIVE) study is an ongoing multicenter cohort study to map patient-reported outcomes (PROs) in Dutch incident KTRs (20). The study was initiated in Leiden University Medical Center (LUMC) in April 2019 and hereafter joined by Maastricht University Medical Center (MUMC) from January 2021 onwards. A signed informed consent form was obtained prior to participation from all participating KTRs. The POSITIVE study was approved by the institutional review board for non-WMO research (i.e., research not subjected to the Medical Research Involving Human Subjects Act) in both centers and complied with the national guidelines for medical scientific research (21). This specific analysis using the POSITIVE data was also approved by the scientific committee of the Clinical Epidemiology Department in LUMC. Patients were invited to participate in this study if they were admitted for kidney transplantation and: 1) were older than 18 years, 2) had no cognitive impairment as determined by patients’ medical history or healthcare professionals’ opinion, and 3) had sufficient understanding of the Dutch language to complete the questionnaires. The invited patients received information about the study’s aim, procedure, and confidentiality; an informed consent form; and a baseline questionnaire. After providing informed consent, patients filled in the first questionnaire during their hospitalization for kidney transplantation. Afterwards, the KTRs received the questionnaires at 6 weeks, 3 months, 6 months, 1 year, and 2 years after kidney transplantation. For the follow-up measurement, an invitation email was sent to patients 1 week before the scheduled time point to fill out the questionnaire and a reminder email was sent if no response was received. The PROs of interest included: HRQOL, symptom experience (i.e., occurrence and burden), and illness perceptions. The estimated average time to finish the questionnaire was approximately 20 min. As the follow-up of the POSITIVE study is still ongoing, this analysis only used the available PROs collected at transplantation (T0) and 6 weeks after kidney transplantation (T1).

### HRQOL

Generic HRQOL was measured using the 12-item Short-Form Health Survey version 2 (SF-12 v2), from which the physical component summary (PCS) score and the mental component summary (MCS) score were derived, indicating physical and mental HRQOL, respectively. PCS consists of four domains, namely,: physical functioning, physical role functioning, bodily pain, and general health; and MCS consists of the following four domains: vitality, social role functioning, emotional role functioning, and mental health. The SF-12 v2 has a recall time of 1 week (22). Following the SF-12 scoring algorithm and to facilitate interpretation and comparison with other studies, norm-based scoring was applied using standardization to the US population with a mean of 50 and a standard deviation of 10, with higher scores indicating better HRQOL (23).

### Symptom Experience (Occurrence and Burden)

Symptom occurrence and burden were measured using the combination of two questionnaires: Dialysis Symptom Index (DSI) (24) and Modified Transplant Symptom Occurrence and Symptom Distress Scale-59 Items Revised (MTSOSD-59r) (4) to cover both CKD-related and immunosuppressants-related symptoms. The DSI was selected as this questionnaire is—like the SF-12—part of routine Dutch dialysis care and the patient-reported outcome measures (PROMs) registry in nephrology care, hereby facilitating comparison across treatment modalities and different stages of CKD (20, 25). Moreover, previous research supports using the DSI in KTRs (26). As there is a considerable overlap between the DSI and the MTSOSD-59r, we chose to only keep the treatment-related symptoms from the MTSOSD-59r (i.e., Immunosuppression-related side effect). After removing duplicate items, sixty-one symptoms were left in the combined questionnaire, comprising 30 DSI-items and 31 MTSOSD-59r-items, with an open-ended question to add 3 additional symptoms. The occurrence of each symptom was measured using binary response options (“yes” and “no”) and a “total number of symptoms” sum score (range: 0–64) was calculated. The burden of each symptom was measured using a 5-point scale ranging from 0 “not distressing at all” to 4 “terribly distressing.” A “total symptom burden” sum score (range:0–256) was calculated by adding up the response from all items. The recall time of this combined questionnaire is 1 week.

### Illness Perceptions

The following eight illness perceptions were measured using single items on a 0-to-10 response scale using the Brief Illness Perception Questionnaire (Brief-IPQ): consequences, timeline, personal control, treatment control, illness identity, concern, illness coherence, and emotional response (27). Like other studies (14), we omitted illness perception “cause” as the cause of kidney disease is very heterogeneous. We recoded the scores for three illness perceptions (i.e., personal control, treatment control, and illness coherence) to facilitate interpretation so that a higher score always indicated stronger negative illness perceptions. Following the B-IPQ instructions, we calculated an overall score for illness perceptions by adding up the scores of all eight perceptions, resulting in a “total illness perceptions score” ranging from 0 “patients perceive their kidney disease as a benign condition” to 80 “patients perceive their kidney disease as a threatening condition” (28, 29). The Cronbach’s alpha value of the total illness perceptions score in our study population was 0.7, indicating a good and sufficient internal consistency to use this total illness perceptions score (22).

### Covariates

Patients’ demographic and clinical characteristics at transplantation were retrieved from their medical records, including age at transplantation, sex, socioeconomic status (SES), PKD, comorbidities, and donor type. The SES of study participants was obtained by linking the four digits of their postcode with the latest SES scores reported by the Netherlands Institute for Social Research. The postcode was considered a proxy of patients SES covering income, educational background and position in the labor market (30). PKD included four categories following the European Renal Association codes: glomerulonephritis, diabetes mellitus, hypertension or renal vascular disease, and other PKDs (31). Comorbidities were defined based on a history of cardiovascular events, cerebrovascular events, and diabetes mellitus. Donor type included living and deceased donors.

### Statistical Analysis

Continuous variables were presented as mean with standard deviation (SD) or median with interquartile range (IQR) depending on their distribution. Categorical variables were presented as counts (percentages). This analysis used symptom experience (i.e., occurrence and burden) measured at T0 and illness perceptions and HRQOL measured at T1 to achieve a temporal sequence of the variables being studied. Patients who responded at T0 and T1 were included in the analysis. HRQOL scores at T1 were calculated and compared to HRQOL at T0 and HRQOL of the general Dutch population (32). The means of the number of symptoms and symptom burden were calculated. A “top 10” list of symptoms in terms of occurrence and burden was presented to describe the symptom experience in the study population at transplantation.

**FIGURE 1:**
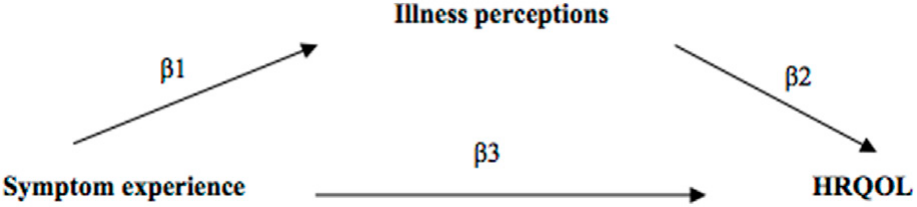
Hypothesized pathways of the mediation effect of illness perceptions between symptom experience and HRQOL.

Multivariable linear regression analysis was used to test the impact of symptom occurrence and symptom burden on both physical and mental HRQOL separately and also to conduct the mediation analysis while adjusting for potential baseline confounders. The hypothesized exposure-outcome, exposure-mediator, and mediator-outcome confounders were structured using Directed Acyclic Graphs (Supplementary Figure S2) and included: age, sex, SES, PKD, donor type, and comorbidities. The mediation analysis was conducted using “the product method” with the total illness perceptions score as a mediator (33). The indirect effect, also called the mediation effect, was calculated by multiplying the beta-coefficient (β1) of symptom occurrence or symptom burden when regressing the total illness perceptions score on symptom occurrence or symptom burden, and the beta-coefficient (β2) of the total illness perceptions score when regressing the physical or mental HRQOL on the total illness perceptions score; the total effect equals the sum of the direct effect (β3) and indirect effect (β1*β2) and refers to the impact of symptom occurrence or burden on physical or mental HRQOL (Figure 1) (33). Bootstrapping method was used to calculate the 95% confidence interval (CI) of the mediation effect using the PROCESS macro for SPSS software (34). The exposure-mediator interaction was checked for the mediation analysis.

Missing values were considered missing at random and were imputed with 10-folds multiple imputation (35). The mediation effects in each imputed dataset were pooled using the package “miWQS” following Rubin’s rule in R version 3.6.1. Given the relatively high percentages of missing values in comorbidities and the relatively small sample size, we conducted our main analysis with and without including comorbidities in the multivariable models.

To test the robustness of our results, we conducted two sensitivity analyses: a complete case analysis and analyses with symptom experience measured using the DSI-items and the remaining MTSOSD-59R-items as patients may not have immunosuppressant-related symptoms at transplantation. Finally, baseline characteristics of study participants and non-participants were tabulated to explore the representativeness of our study population. We used SPSS software version 25.0. (IBM, Armonk, NY, United Sates) for all analyses if not indicated otherwise. Statistical significance was determined by a *p*-value <0.05 or when the 95% CI did not contain the null-effect value of “zero.”

## Results

### Patient Characteristics

**FIGURE 2:**
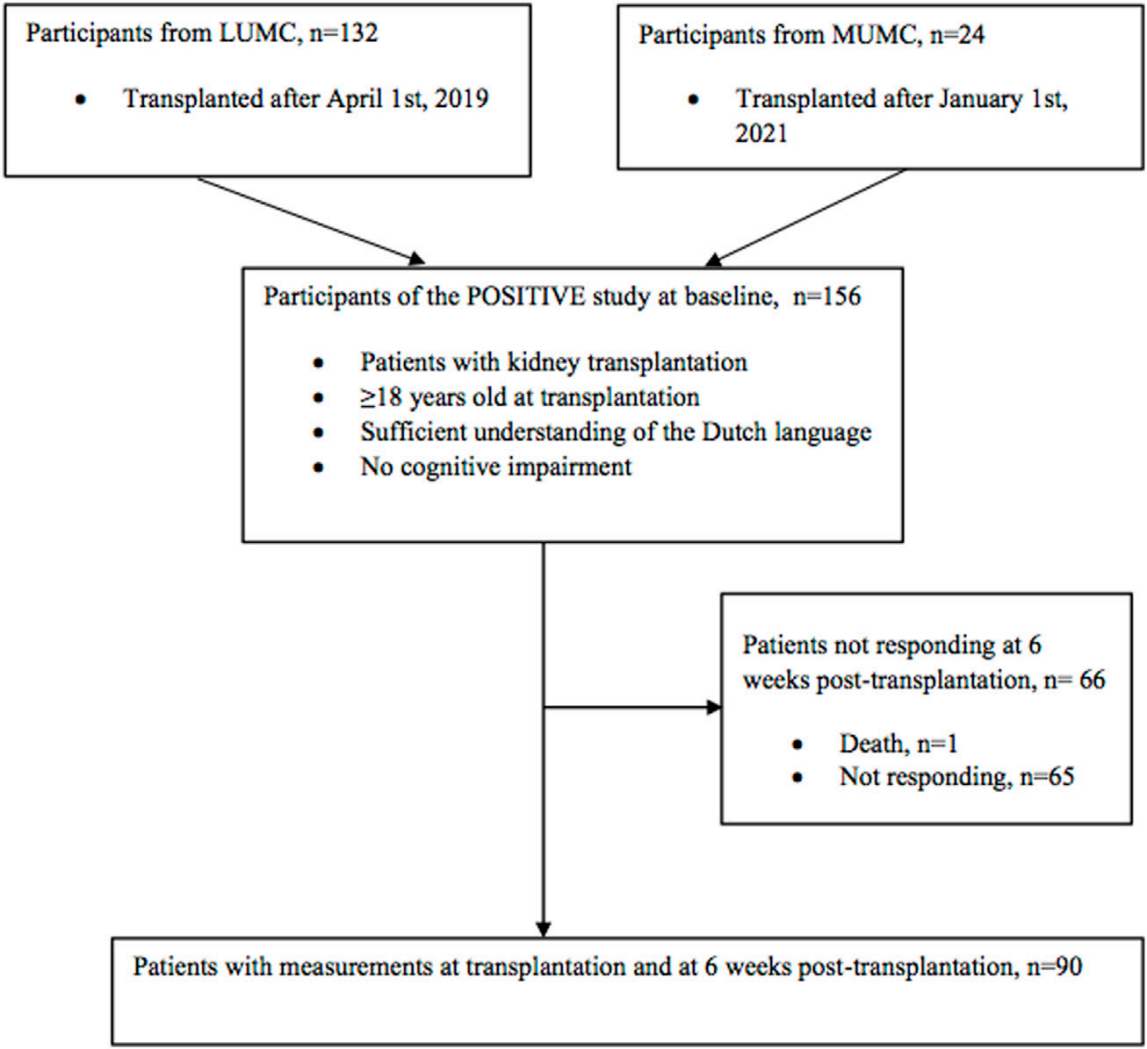
Flow chart of the study population.

Of the 156 KTRs included in our study at transplantation (T0), 90 KTRs (58%) responded at 6 weeks after kidney transplantation (T1) and were included in the main analysis. One patient deceased before the measurement at T1, and 65 (42%) patients did not respond to the follow-up questionnaires (Figure 2). The average time (SD) between the measurement at T0 and T1 was 5.6 (1.9) weeks. The clinical and demographic characteristics of the study population are presented in Table 1. Our population had an average age of 52.5 years (SD, 13.8), 36% were female, 66% received a living donor kidney transplantation, and glomerulonephritis was the most common PKD. Compared to the responders at 6 weeks, non-responders were more likely to have a deceased donor, diabetes mellitus as PKD or comorbidity, and a history of cardiovascular events (Table 1). The participants and non-participants of the study were similar in the following characeristics: age, sex and donor type. Compared to participants, more non-participants had a low SES and diabetes mellitus as PKD and comorbidity (Supplementary Table S1).

**TABLE 1:** Clinical and demographic characteristics of the study population.

Characteristics	T0 (*n* = 156)	Responders at T1 (*n* = 90)	Non-responders at T1 (*n* = 66)
Mean age (SD)	53.3 (13.5)	52.5 (13.8)	54.3 (13.0)
Female, n (%)	56 (36)	32 (36)	24 (36)
SES, n (%)			
Low	25 (16)	14 (16)	11 (17)
Middle	103 (66)	61 (68)	42 (64)
High	26 (17)	15 (17)	11 (17)
Primary kidney disease, n (%)			
Diabetes mellitus	29 (19)	15 (17)	14 (21)
Glomerulonephritis	36 (23)	23 (26)	13 (18)
Renal vascular disease	18 (12)	12 (13)	6 (9)
Other diseases	71 (46)	40 (44)	31 (47)
Donor type, n (%)			
Living donor	89 (57)	59 (66)	30 (46)
Deceased donor	65 (42)	31 (34)	34 (52)
Comorbidities, n (%)^a^			
Diabetes mellitus	18 (12)	8 (9)	10 (15)
Cardiovascular event	24 (15)	7 (8)	17 (26)
Cerebrovascular event	8 (5)	5 (6)	3 (5)

^a^ Missing values: diabetes mellitus, cardiovascular event, cerebrovascular event (baseline: 37.8%, 32.1%, 32.1%; responders: 36.7%, 33.3%, 33.3%.; non-responders: 39.4%, 30.3%, 30.3%). Non-responders had 2% missing values in age, SES, primary kidney disease and donor type. Abbreviations: SES, socioeconomic status; SD, standard deviation.

### Symptom Experience at Kidney Transplantation

The mean number of symptoms (SD) reported by KTRs at T0 was 19 (12) on a 0–64 scale, and the mean symptom burden (SD) was 34 (27) on a 0–256 scale. Table 2 shows the 10 most frequently reported and the most burdensome symptoms. The two ranks had an overlap in the following symptoms: fatigue, lack of energy, difficulty falling asleep, difficulty staying asleep, and decreased appetite. Sex-specific symptoms (i.e., erection problem in males and menstrual problem in females) and difficulty becoming sexually aroused had a lower rank in occurrence but were considered very burdensome. Supplementary Figure S3 shows the occurrence and the mean burden of individual symptoms at T0.

**TABLE 2:** Symptom experience (symptom occurrence and symptom burden) of the study population at T0 (*n* = 90).

	Symptom occurrence	n (%)	Symptom burden	Mean (SD)
Rank (starting from the most reported/burdensome)				
1	Fatigue	76 (86)	Fatigue	2.4 (1.2)
2	Lack of energy	68 (77)	Lack of energy	2.4 (1.1)
3	Difficulty staying asleep	57 (64)	Sex-specific symptom^a^	2.3 (1.2)
4	Increased urge to urinate at night	56 (63)	Difficulty falling asleep	2.2 (1.1)
5	Difficulty falling asleep	47 (53)	Decreased appetite	2.2 (1.2)
6	Decreased appetite	42 (47)	Sweat more	2.1 (1.2)
7	Flatulence	42 (47)	Difficulty staying asleep	2.0 (1.1)
8	Memory problems	42 (47)	Muscle weakness	2.0 (0.9)
9	Difficulty concentrating	41 (47)	Restless legs	2.0 (1.0)
10	Dry skin	41 (46)	Difficulty becoming sexually aroused	2.0 (1.1)
Total score, mean (SD)		19 (12)	Total score, mean(SD)	34 (27)

^a^ Erection problem in males and menstrual problem in females. Five patients with more than 5 missing values in their symptom checklist were excluded from the descriptive statistics in the table. Abbreviations: SD, standard deviation.

### HRQOL at 6 Weeks After Kidney Transplantation

KTRs at T2 reported a mental HRQOL (mean [SD]; 49.9 [10.7]) which was significantly higher than at T0 (44.7 [10.7]) and similar to the general Dutch population (50.2 [9.2]). Physical HRQOL (38.9 [9.1]) was similar to that reported at T0 (39.9 [9.6]) but significantly lower than the general Dutch population (50.6 [9.2]) (Table 3) (32). Scores of the HRQOL-domains *general health*, *vitality*, and *mental health* increased on average by 8.0 (13.0), 6.0(12.9), and 4.1 (12.7) compared to the scores at T0, indicating better general health, more energy, and less mental distress in KTRs at T1; the score for *bodily pain* reduced by −5.2 (11.9), indicating a larger influence of bodily pain on routine activities at T1. No significant changes were found in the other four HRQOL-domains (i.e., *physical function*, *role physical*, *social functioning,* and *role emotional*).

**TABLE 3:** HRQOL at T0 and T1 in comparison to the Dutch general population.

HRQOL score^a^	At T0 (*n* = 82)	At T1 (*n* = 89)	Dutch GP (*n* = 2013) (32)	Mean difference between different time points or groups					
				T1-T0	*p*-value^b^	Dutch GP-T0	*p*-value^c^	Dutch GP- T1	*p*-value^c^
PF	41.0 (11.3)	40.2 (10.9)	—	−0.6 (12.0)	0.63	—	—	—	—
RP	36.2 (10.1)	36.3 (8.7)	—	0.1 (10.6)	0.92	—	—	—	—
BP	49.6 (10.5)	44.1 (11.4)	—	−5.2 (11.9)	<0.001	—	—	—	—
GH	36.6 (11.1)	44.6 (10.3)	—	8.0 (13.3)	<0.001	—	—	—	—
VT	43.7 (10.4)	49.5 (10.9)	—	6.0 (12.9)	<0.001	—	—	—	—
SF	38.6 (13.9)	40.5 (12.2)	—	2.1 (16.5)	0.25	—	—	—	—
RE	40.4 (12.8)	42.7 (11.5)	—	2.7 (14.0)	0.08	—	—	—	—
MH	48.7 (10.7)	52.5 (10.5)	—	4.1 (12.7)	0.01	—	—	—	—
PCS	39.9 (9.6)	38.9 (9.1)	50.6 (9.2)	−1.2 (9.5)	0.28	10.7 (1.0)	<0.001	11.7 (1.0)	<0.001
MCS	44.7 (10.7)	49.9 (10.7)	50.2 (9.2)	5.7 (12.5)	<0.001	5.5 (1.0)	<0.001	0.3 (1.0)	0.76

Abbreviations: BP, bodily pain; GH, general health; GP, general population; KT, kidney transplantation; MCS, mental component scale; MH, mental health; PCS, physical component scale; PF, physical functioning; RE, role emotional; RP, role physical; SF, social functioning; VT, vitality.

^a^ All HRQOL scores and their mean differences were reported as mean and standard deviation.

^b^ The *p*-value was calculated using paired sample *t*-test, and 82 patients without missing values in the 12-item Short Form Survey at KT and 6 weeks after KT were included for this comparison.

^c^ The *p*-value was calculated using independent sample *t*-test.

### Illness Perceptions at 6 Weeks After Kidney Transplantation

The individual and total mean (SD) illness perceptions scores reported by KTRs at T1 are shown in Table 4. Individual illness perceptions scores were measured on a scale from 0-to-10 (27). The study population reported a good understanding of their kidney disease (*illness coherence*; 1.9 [2.0]). They considered their kidney disease a chronic condition (*timeline*; 7.6 [3.4]) that negatively influences their life (*consequence*; 6.2 [3.0]). They reported a moderate level of worrying (*concern*; 4.8 [2.8]) and emotional distress due to their kidney disease (*emotional response*; 3.2 [2.7]). They believed that a moderate amount of symptoms can be attributed to their kidney disease (*illness identity*; 4.5 [2.9]), and they believed to a great extent that the treatment they receive (e.g., kidney transplantation) can effectively control their kidney disease (*treatment control*; 1.8 [2.2]), but to a lesser extent that they can control the disease themselves (*personal control*; 3.8 [2.5]). The mean total illness perceptions score (SD) was 34.1 (12.3) on a scale from 0-to-80, indicating that patients perceived their kidney disease as a threatening condition at a moderate level.

**TABLE 4:** Illness perceptions of the study population at T1 (*n* = 90).

Illness perception	Mean (SD)	A higher score indicates patients believe to a greater extent that…
Consequences	6.2 (3.0)	their kidney disease has more negative consequences upon their life
Timeline	7.6 (3.4)	their kidney disease lasts for a longer time
Personal control	3.8 (2.5)	their kidney disease cannot be effectively controlled by themselves
Treatment control	1.8 (2.2)	their kidney disease cannot be effectively controlled by their treatment
Illness identity	4.5 (2.9)	their kidney disease causes more symptoms
Concern	4.8 (2.8)	their kidney disease causes greater worries about their health
Illness coherence	1.9 (2.0)	they do not understand their kidney disease
Emotional response	3.6 (2.7)	their kidney disease causes more emotional distress
Total score^a^	34.1 (12.3)	their kidney disease is a more threatening condition

^a^ Total score was measured on a 0-to-80 scale and the domain scores on a 0-to-10 scale. One patient with missing values in the illness perception questionnaire was excluded from the descriptive statistics. Abbreviations: SD, standard deviation.

### Impact of Symptom Experience on HRQOL After Kidney Transplantation

Table 5 presents the impact of KTRs’ symptom experience at T0 on their physical and mental HRQOL at T1 (i.e., total effect) and the mediation effect of illness perceptions (i.e., indirect effect). The unadjusted analyses showed that mental and physical HRQOL reduced by −0.17 (95%CI: −0.33, −0.01) and −0.24 (95%CI: −0.42, −0.05) with each additional symptom, respectively. After adjusting for potential baseline confounders with and without comorbidities, the decline in mental HRQOL with each additional symptom remained statistically significant and was −0.23 (95%CI: −0.42, −0.04) and −0.24 (95%CI: −0.42, −0.05), respectively. The unadjusted and adjusted analysis showed a statistically insignificant decrease in mental and physical HRQOL with an increase in symptom burden.

**TABLE 5:** Impact of symptom experience at T0 on HRQOL at T1 and the mediation effect of illness perception (*n* = 90).

	Estimates	Crude β (95%CI)	*p*-value	Adjusted β (95%CI)^a^	*p*-value	Adjusted β (95%CI)^b^	*p*-value
Number of symptoms and HRQOL							
PCS	Total effect^c^	−0.17 (−0.33, −0.01)	0.04	−0.16 (−0.32, 0.01)	0.06	−0.15 (−0.31, 0.02)	0.09
	Direct effect	−0.10 (−0.26, 0.06)	0.20	−0.08 (−0.24, 0.09)	0.35	−0.09 (−0.26, 0.07)	0.27
	Indirect effect	−0.07 (−0.13, −0.01)		−0.06 (−0.13, 0.003)		−0.05 (−0.12, 0.01)	
MCS	Total effect	−0.24 (−0.42, −0.05)	0.01	−0.24 (−0.42, −0.05)	0.01	−0.23 (−0.42, −0.04)	0.02
	Direct effect	−0.10 (−0.26, 0.07)	0.25	−0.13 (−0.28, 0.03)	0.12	−0.13 (−0.19, 0.03)	0.11
	Indirect effect	−0.14 (−0.25, −0.04)		−0.11 (−0.22, 0.004)		−0.10 (−0.21, 0.01)	
Symptom burden and HRQOL							
PCS	Total effect	−0.06 (−0.12, 0.02)	0.12	−0.05 (−0.12, 0.02)	0.19	−0.04 (−0.12, 0.03)	0.26
	Direct effect	−0.03 (−0.10, 0.04)	0.44	−0.02 (−0.09, 0.05)	0.50	−0.02 (−0.09, 0.05)	0.56
	Indirect effect	−0.03 (−0.05, −0.003)		−0.03 (−0.05, 0.002)		−0.02 (−0.05, 0.01)	
MCS	Total effect	−0.07 (−0.15, 0.01)	0.08	−0.07 (−0.15, 0.01)	0.10	−0.07 (−0.15, 0.02)	0.11
	Direct effect	−0.02 (−0.09, 0.05)	0.63	−0.02 (−0.09, 0.04)	0.49	−0.03 (−0.10, 0.04)	0.45
	Indirect effect	−0.06 (−0.10, −0.01)		−0.04 (−0.09, 0.003)		−0.04 (−0.08, 0.04)	

The *p*-values of the interaction term between symptom experience and illness perceptions ranged from 0.13 to 0.98. Abbreviations: CI, confidence interval; HRQOL, health-related quality of life; MCS, mental component scale; PCS, physical component scale; RR, risk ratio; SD, standard deviation.

^a^ The adjusted variables include age, sex, SES, primary kidney disease, and donor type.

^b^ The adjusted variables include age, sex, SES, primary kidney disease donor type, and comorbidities.

^c^ The total effect is the sum of the direct and indirect effects.

### Mediation Effect of Illness Perceptions

The unadjusted mediation effect of illness perceptions was −0.07 (95%CI: −0.13, −0.01) between the number of symptoms and physical HRQOL; −0.14 (95%CI: −0.25, −0.04) between the number of symptoms and mental HRQOL; −0.03 (95%CI: −0.05, −0.003) between symptom burden and physical HRQOL; and −0.06 (95%CI: −0.10, −0.01) between symptom burden and physical HRQOL (Table 5). The negative mediation effects indicate corresponding reductions in HRQOL due to the increased strength of negative illness perceptions following each additional symptom or each point increase in symptom burden score. After adjustment with or without comorbidities, β-coefficients remained similar or slightly changed; the 95%CI became broader than the unadjusted results with the upper confidence limit larger than but close to the no-effect value of “zero.”

### Sensitivity Analysis

Results from the complete case analysis (*n* = 87) and the analyses with symptom experience measured using the DSI-items and the rest of the items, supported results from the main analysis (Supplementary Tables S2–S4).

## Discussion

Our study showed a considerable number of symptoms and a moderate level of symptom burden at transplantation in Dutch KTRs. Mental HRQOL 6 weeks after kidney transplantation was higher than HRQOL at transplantation and became comparable to HRQOL in the general Dutch population, whereas physical HRQOL remained unchanged compared to HRQOL at transplantation and was significantly lower than HRQOL in the general Dutch population. The number of symptoms had a significant effect on mental HRQOL and a borderline significant effect on physical HRQOL, while the effect of symptom burden on HRQOL was small and not significant. Furthermore, our results suggest that illness perceptions mediate the effects of symptom experience on both mental and physical HRQOL in KTRs in the short term after kidney transplantation.

Our study population experienced, on average, nineteen out of sixty-four symptoms at transplantation. The number of symptoms in our study is larger than seven out of twenty-six detected by a study in prevalent KTRs in the UK (3) and ten out of thirty in Dutch dialysis patients (25). The proportions of symptoms reported by patients could be considered similar in the three studies, suggesting that these patient groups may experience a comparable number of symptoms. However, no solid conclusion can be drawn as different questionnaires were used. Notably, the most frequently experienced symptoms in our study were similar to those from the previous studies, with the top three being identical, namely,: fatigue, lack of energy, and sleep problems (3, 25). Fatigue and lack of energy were also the most burdensome symptoms in our study population, as well as prevalent KTRs in the UK (3).

KTRs in our study had similar mental HRQOL but lower physical HRQOL at 6 weeks after transplantation than the general Dutch population (32). Previous studies have reported similar results in KTRs (36, 37). KTRs 6 weeks after transplantation had similar physical HRQOL and improved mental HRQOL than themselves at transplantation. The stable physical HRQOL can be a trade-off between improved general health and increased impact of bodily pain on daily activities that is most likely due to the recent surgical procedure. The improved mental HRQOL in our study population was a result of the improvement in the domains *vitality* and *mental health* after transplantation, suggesting that KTRs became more energetic and had less mental distress. Previous studies echo this finding showing more physical activities and less depressive symptoms in KTRs than dialysis patients (38, 39).

Our study population believed to a moderate extent that their kidney disease is a threatening condition. Specifically, patients believed to a great extent that they understand their kidney disease and that their treatment can control their kidney disease. However, patients also believed to a great extent that their kidney disease has many negative consequences upon their lives. The separate illness perceptions scores in our study population are comparable to those in another Dutch KTRs cohort, except for *illness identity*: our study population reported a higher score, indicating that patients attributed more symptoms to their kidney disease (40). This difference could be explained by the different time after kidney transplantation when the measurements were conducted and the 14% more KTRs with deceased donors in our study population who are more likely to have comorbidities (40, 41).

Our analysis indicates that the number of symptoms impacted HRQOL in KTRs. This finding is in accordance with results from a previous study in Dutch CKD patients prior to kidney replacement therapy, showing lower HRQOL in patients with more symptoms(5). The impact on HRQOL with each increment in symptom burden score was statistically insignificant, which is most likely due to our small sample size. Furthermore, our analysis revealed mediation effects of illness perceptions with 0 being the upper limit of its 95% CI after adjustment without comorbidities. Based on literature (42) and the significant mediation effects in the complete case analysis consisting 97% of the study population (Supplementary Table S2), our results could indicate that worse symptom experience (i.e., more symptoms or a higher symptom burden) at transplantation leads to unhelpful illness perceptions, which consequently leads to lower HRQOL after kidney transplantation. A previous study found the same mechanism in Dutch patients with irritable bowel syndrome (43). After adjusting for comorbidities, the mediation effects remained similar or became slightly smaller. However, the 95% CI became wider due to our relatively small sample size and the large percentage of missing values in comorbidities despite being imputed. Future studies with a larger sample size are necessary to confirm our findings.

Our results suggest the potential benefit of active symptom management among KTRs regarding HRQOL. Actively treating symptoms requires structural identification of patients’ symptom experience. Studies have shown positive results of clinically implementing symptom-checklists for this purpose (25, 44). Moreover, our findings support the use of Leventhal’s CSM of self-regulation (9) to explain the impact of symptom experience on HRQOL in KTRs and suggest the potential of illness perceptions as interventional targets to reduce the impact of symptom experience on HRQOL. Please note that we measured HRQOL 6 weeks after transplantation; patients’ HRQOL during the first 6 weeks could be influenced by many other factors (e.g., surgery-related complications or withdrawal of dialysis), which could diminish the impact of symptom experience at transplantation on HRQOL 6 weeks after transplantation. Despite the relatively small impact of symptom experience on HRQOL detected in our analysis, our results suggest a mediation effect of illness perceptions, and we speculate that the impact is larger in KTRs at a more stable stage for the reason mentioned above. Therefore, modifying unhelpful illness perceptions could potentially alleviate the negative influence of symptom experience in HRQOL to a greater extent in stable patients. Furthermore, unhelpful illness perceptions are common and identified as important risk factors for health outcomes among patients in different CKD stages, including HRQOL, kidney function, or graft function (12, 14, 15). Moreover, past research has shown that unhelpful illness perceptions are modifiable by means of psycho-educational support strategies and can lead to improved coping behaviors and health outcomes (17, 18, 45). Future studies in KTRs are needed to: 1) further explore the role of illness perceptions in the relationship between symptom experience and HRQOL at a stable stage to provide further information for clinical practice, 2) explore the mediation effect of individual illness perceptions to provide more precise intervention targets, and 3) explore whether support strategies targeting unhelpful perceptions indeed lead to improved outcomes.

Our study has several strengths. First, our study generates new insights into patient-reported outcomes shortly after kidney transplantation. Second, our study is the first to explore the potential mechanism of the impact of symptom experience on HRQOL in KTRs and herein examine the potential of modifying illness perceptions in order to improve impaired HRQOL due to symptoms. Third, our longitudinal study is more appropriate to evaluate the influence of symptom experience on HRQOL than a cross-sectional study. Our study also has limitations. First, as mentioned above, a number of factors can influence patients’ HRQOL shortly after transplantation, and the impact of symptom experience at transplantation on HRQOL may not be dominant. Data with regard to surgery-related complications and lifestyle change (e.g., dialysis withdrawal) may be collected in future study to better explain the HRQOL change in this period. Nevertheless, we detected a significant impact of symptom number on HRQOL. However, our sample size was most likely insufficient to detect the relatively small effect of symptom burden on HRQOL. Please note that the symptom burden score ranges from 0 to 256, which still has the potential to influence HRQOL largely despite a small effect of one increment in symptom burden score on HRQOL. Second, the percentage of non-responders at 6 weeks after kidney transplantation was relatively high (42.3%), which could influence the representativeness of our study population or introduce selection bias. The non-responders in our study were older and had more often diabetes as PKD, more comorbidities, and more often deceased donors. Finally, this observational study cannot prove causality. In addition, due to the limited sample size, we did not adjust all factors that were suggestive of patient’s health at transplantation, such as time on dialysis or preemptive transplantation or not. Instead, donor type was adjusted and considered a proxy for these factors, which could cause residual confounding.

In conclusion, symptom experience at transplantation can influence HRQOL shortly after kidney transplantation, and this influence is partially mediated by patients’ illness perceptions, suggesting the potential benefit of active symptom management and modifying patients’ unhelpful perceptions in optimizing post-transplant HRQOL. Future studies in KTRs at different stages after kidney transplantation are needed to confirm our findings.

## Data Availability

The datasets presented in this article are not readily available because the data are collected for this specific study. Requests to access the datasets should be directed to FD, f.w.dekker@lumc.nl.

## Abbreviations

B-IPQ, the brief illness perception questionnaire; CI, confidence interval; CKD, chronic kidney disease; CSM, Leventhal’s common-sense model of self-regulation; DSI, dialysis symptom index; HRQOL, health-related quality of life; KTRs, kidney transplant recipients; LUMC, Leiden University Medical Center; MCS, mental component summary; MUMC, Maastricht University Medical Center; MTSOSD-59r, the modified transplant symptom occurrence and symptom distress scale-59 items revised; PCS, physical component summary; PKD, primary kidney disease; POSITIVE, the Patient-reported Outcomes In kidney Transplant recipients: Input of Valuable Endpoints Study; PROMs, patient-reported outcome measures; SES, socioeconomic status; SD, standard deviation; SF-12, the 12-item short-from health survey; STROBE, the strengthening the reporting of observational studies in epidemiology guideline.
