# Supplementary material for: Understanding Health-Related Quality of Life in Kidney Transplant Recipients: The Role of Symptom Experience and Illness Perceptions
Source: Transpl Int. 2023 Apr 13;36:10837. doi: 10.3389/ti.2023.10837 (PMC10134035; doi:10.3389/ti.2023.10837)
Supplement: Supplementary file 1 [file DataSheet1.pdf]

Supplementary file

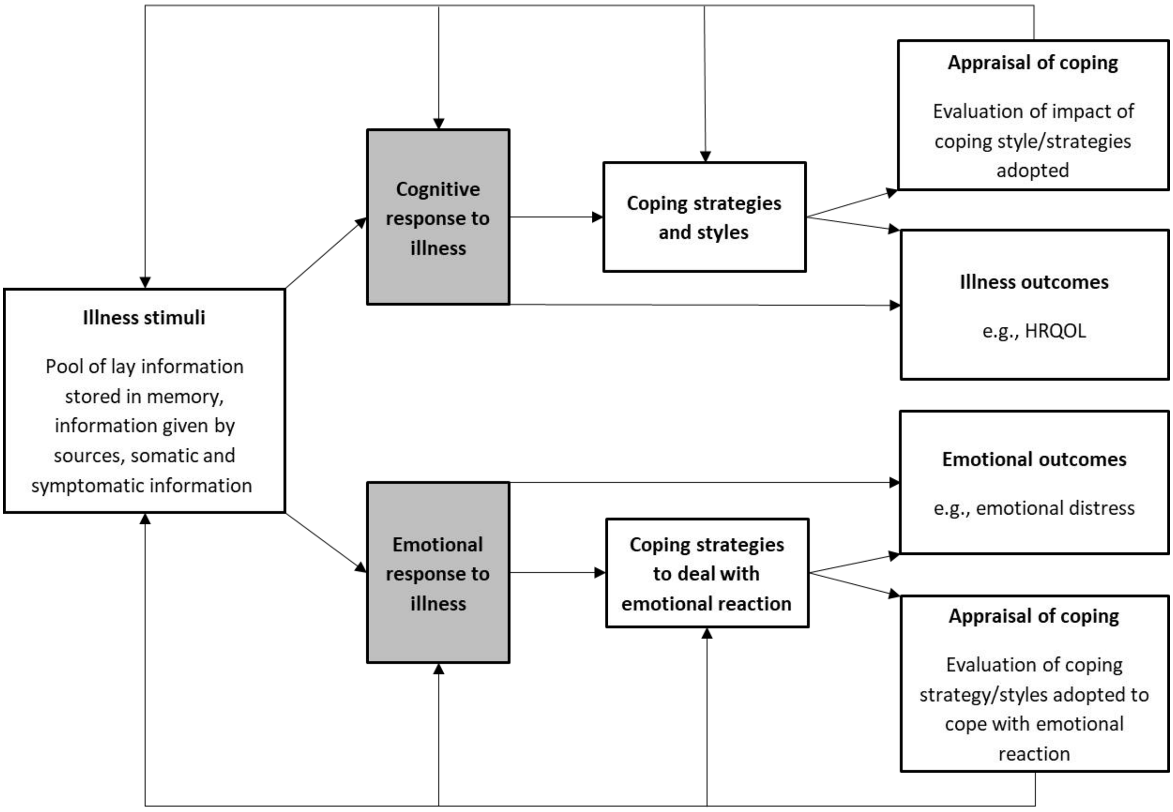

Figure S1. Visual representation of Leventhal's Common Sense Model of self-regulation (adapted from Hagger & Orbell, 2003). The boxes in grey represent the 'illness perceptions'.

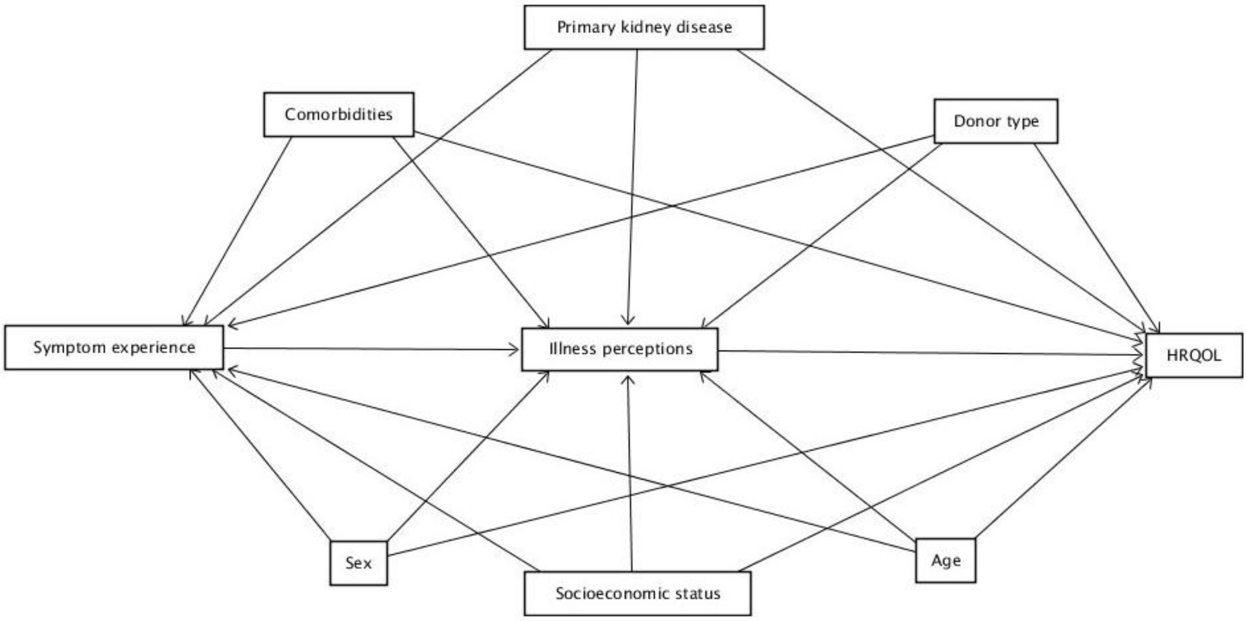

Figure S2. Directed acyclic graphs (DAGs) structured the potential exposure-outcome, exposure-mediator, and mediator-outcome confounders. Abbreviations: HRQOL, health-related quality of life.

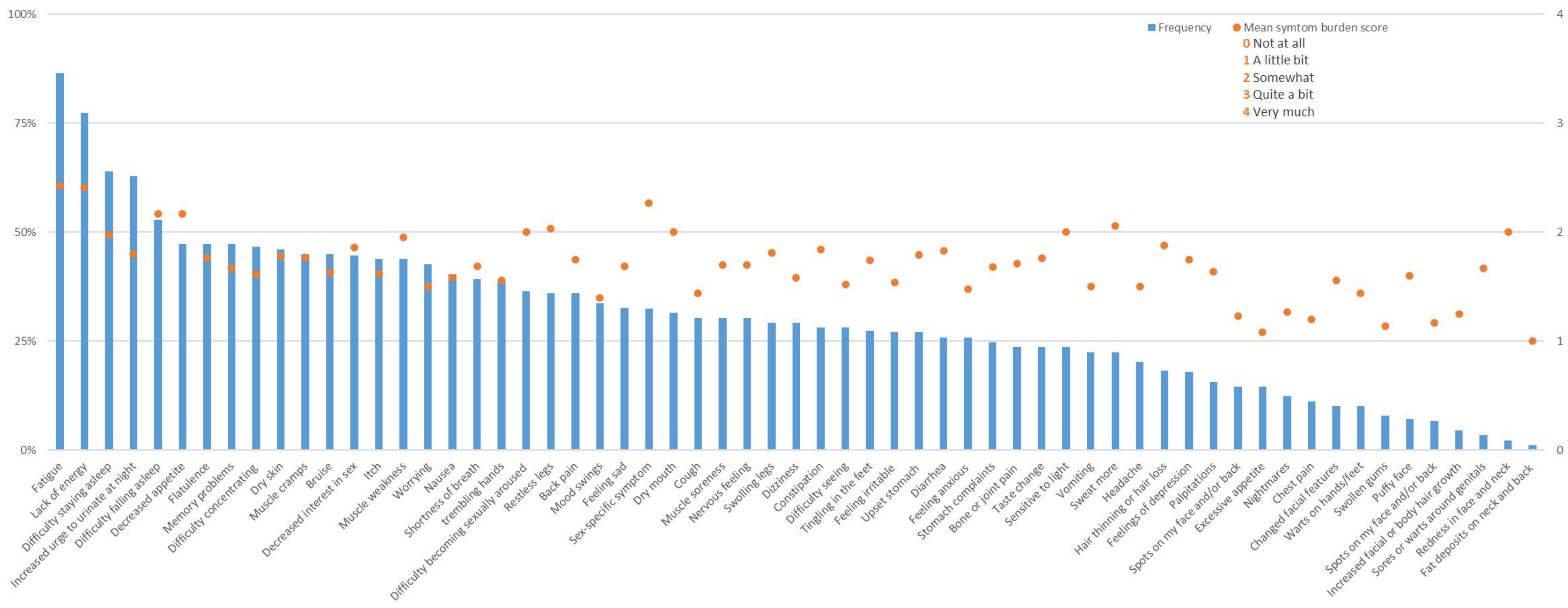

Figure S3. Symptom experience of the study population at T0 (n=90). One patient with more than 5 missing values in their symptom checklist was excluded from the descriptive statistics in the figure.

Table S1. Clinical and demographic characteristics of participants and non-participants.

Characteristics	Participants (n=156)	Non-participants (n=243)
**Mean age (SD)[a]**	53.3 (13.5)	53.1 (14.5)
**Female, n(%)**	56 (36)	94 (39)
**SES, n(%)[a]**		
Low	25 (16)	57 (24)
Middle	103 (66)	134 (55)
High	26 (17)	46 (19)
**Primary kidney disease, n(%)[a]**		
Diabetes mellitus	29 (19)	70 (29)
Glomerulonephritis	36 (23)	48 (20)
Renal vascular disease	18 (12)	14 (6)
Other diseases	71 (46)	111 (45)
**Donor type, n(%)[a]**		
Living donor	89 (57)	134 (55)
Deceased donor	65 (42)	109 (45)
**Comorbidities, n(%)[a]**		
Diabetes mellitus	18 (12)	42 (17)
Cardiovascular event	24 (15)	35 (14)
Cerebrovascular event	8 (5)	13 (5)

[a]Missing values: diabetes mellitus, cardiovascular event, cerebrovascular event (participants: 37.8%, 32.1%, 32.1%; non-participants: 47.7%, 44.0%, 44.4%). Participants had 1% missing values in age, SES, primary kidney disease and donor type. Non-participants had 2% missing values in SES. Abbreviations: SES, socioeconomic status; SD, standard deviation.

Table S2. Impact of symptom experience at T0 on HRQOL at T1 and the mediation effect of illness perceptions (n=87, complete case analysis).

	Estimates	Crude β (95%CI)	P-value	Adjusted β (95%CI)[a]	P-value
**Number of symptoms and HRQOL**					
**PCS**	**Total effect**[b]	-0.17 (-0.34, -0.01)	0.04	-0.15 (-0.32, 0.03)	0.09
	**Direct effect**	-0.10 (-0.26, 0.07)	0.24	-0.08 (-0.24, 0.09)	0.35
	**Indirect effect**	-0.07 (-0.15, -0.02)		-0.07 (-0.14, -0.005)	
**MCS**	**Total effect**	-0.24 (-0.43, -0.05)	0.01	-0.24 (-0.43, -0.05)	0.02
	**Direct effect**	-0.10 (-0.26, 0.07)	0.25	-0.12 (-0.28, 0.04)	0.14
	**Indirect effect**	-0.15 (-0.26, -0.05)		-0.11 (-0.23, -0.01)	
**Symptom burden and HRQOL**					
**PCS**	**Total effect**	-0.05 (-0.13, 0.02)	0.13	-0.04 (-0.12, 0.03)	0.24
	**Direct effect**	-0.02 (-0.09, 0.04)	0.48	-0.02 (-0.09, 0.05)	0.63
	**Indirect effect**	-0.03 (-0.06, -0.01)		-0.03 (-0.06, -0.001)	
**MCS**	**Total effect**	-0.08 (-0.16, 0.01)	0.07	-0.07 (-0.15, 0.02)	0.11
	**Direct effect**	-0.02 (-0.09, 0.05)	0.63	-0.02 (-0.09, 0.05)	0.54
	**Indirect effect**	-0.06 (-0.10, -0.02)		-0.04 (-0.10, -0.002)	

The p-values of the interaction term between symptom experience and illness perception ranged from 0.13 to 0.97. Abbreviations: CI, confidence interval; HRQOL, health-related quality of life; MCS, mental component scale; PCS, mental component scale; RR, risk ratio; SD, standard deviation.

[a]The adjusted variables include age, sex, socioeconomic status (continuous scale), primary kidney disease, and donor type.

[b]The total effect is the sum of the direct and indirect effects.

Table S3. Impact of symptom experience (based on the DSI-items only) at T0 on HRQOL at T1 and the mediation effect of illness perceptions (n=90).

	Estimates	Crude β (95%CI)	P-value	Adjusted β (95%CI)[a]	P-value	Adjusted β (95%CI)[b]	P-value
**Number of symptoms and HRQOL**							
**PCS**	**Total effect[c]**	-0.28 (-0.55, -0.01)	0.04	-0.25 (-0.52, 0.02)	0.07	-0.23 (-0.51, 0.05)	0.10
	**Direct effect**	-0.16 (-0.43, 0.11)	0.24	-0.14 (-0.41, 0.12)	0.28	-0.13 (-0.40, 0.14)	0.29
	**Indirect effect**	-0.12 (-0.21, -0.02)		-0.11 (-0.22, 0.002)		-0.10 (-0.21, 0.02)	
**MCS**	**Total effect**	-0.46 (-0.76, -0.15)	0.004	-0.43 (-0.72, -0.13)	0.01	-0.41 (-0.72, -0.11)	0.01
	**Direct effect**	-0.22 (-0.48, 0.05)	0.11	-0.25 (-0.50, 0.01)	0.06	-0.24 (-0.51, 0.02)	0.07
	**Indirect effect**	-0.24 (-0.41, -0.07)		-0.18 (-0.36, -0.0002)		-0.17 (-0.36, 0.02)	
**Symptom burden and HRQOL**							
**PCS**	**Total effect**	-0.09 (-0.20, 0.03)	0.15	-0.08 (-0.19, 0.04)	0.22	-0.07 (-0.19, 0.06)	0.29
	**Direct effect**	-0.04 (-0.15, 0.07)	0.48	-0.03 (-0.15, 0.08)	0.56	-0.03 (-0.15, 0.09)	0.63
	**Indirect effect**	-0.05 (-0.09, -0.01)		-0.04 (-0.09, 0.004)		-0.04 (-0.08, 0.01)	
**MCS**	**Total effect**	-0.13 (-0.27, 0.003)	0.05	-0.12 (-0.25, 0.01)	0.08	-0.11 (-0.25, 0.03)	0.12
	**Direct effect**	-0.04 (-0.16, 0.07)	0.47	-0.05 (-0.16, 0.07)	0.41	-0.04 (-0.16, 0.07)	0.46
	**Indirect effect**	-0.09 (-0.16, -0.02)		-0.07 (-0.14, 0.01)		-0.07 (-0.14, 0.01)	

The p-values of the interaction term between symptom experience and illness perception ranged from 0.13 to 0.98. Abbreviations: CI, confidence interval; DSI, the dialysis symptom index questionnaire; HRQOL, health-related quality of life; MCS, mental component scale; PCS, mental component scale; RR, risk ratio; SD, standard deviation.

[a]The adjusted variables include age, sex, SES, primary kidney disease, and donor type.

[b]The adjusted variables include age, sex, SES, primary kidney disease donor type, and comorbidities.

[c]The total effect is the sum of the direct and indirect effects.

Table S4. Impact of symptom burden (based on the remaining symptoms) at T0 on HRQOL at T1, and the mediation effect of illness perceptions (n=90).

	Estimates	Crude β (95%CI)	P-value	Adjusted β (95%CI)[a]	P-value	Adjusted β (95%CI)[b]	P-value
**Number of symptoms and HRQOL**							
**PCS**	**Total effect**[c]	-0.35 (-0.71, 0.01)	0.06	-0.32 (-0.71, 0.06)	0.10	-0.30 (-0.70, 0.11)	0.15
	**Direct effect**	-0.21 (-0.57, 0.14)	0.24	-0.20 (-0.57, 0.17)	0.30	-0.19 (-0.57, 0.20)	0.34
	**Indirect effect**	-0.14 (-0.27, -0.01)		-0.12 (-0.27, 0.02)		-0.11 (-0.26, 0.04)	
**MCS**	**Total effect**	-0.38 (-0.81, 0.04)	0.08	-0.40 (-0.83, 0.02)	0.06	-0.38 (-0.82, 0.07)	0.10
	**Direct effect**	-0.09 (-0.44, 0.27)	0.64	-0.19 (-0.55, 0.18)	0.31	-0.18 (-0.56, 0.19)	0.34
	**Indirect effect**	-0.30 (-0.54, -0.06)		-0.22 (-0.47, 0.04)		-0.2 (-0.46, 0.07)	
**Symptom burden and HRQOL**							
**PCS**	**Total effect**	-0.13 (-0.28, 0.03)	0.12	-0.12 (-0.28, 0.05)	0.17	-0.11 (-0.28, 0.06)	0.21
	**Direct effect**	-0.07 (-0.22, 0.09)	0.40	-0.06 (-0.22, 0.09)	0.43	-0.06 (-0.22, 0.10)	0.46
	**Indirect effect**	-0.06 (-0.11, 0.004)		-0.05 (-0.11, 0.01)		-0.05 (-0.11, 0.02)	
**MCS**	**Total effect**	-0.13 (-0.32, 0.05)	0.16	-0.13 (-0.32, 0.06)	0.17	-0.12 (-0.31, 0.07)	0.23
	**Direct effect**	-0.01 (-0.17, 0.15)	0.90	-0.04 (-0.20, 0.12)	0.62	-0.03 (-0.20, 0.13)	0.68
	**Indirect effect**	-0.12 (-0.22, -0.02)		-0.09 (-0.2, 0.02)		-0.08 (-0.20, 0.03)	

The p-values of the interaction term between symptom experience and illness perception ranged from 0.13 to 0.98. Abbreviations: CI, confidence interval; HRQOL, health-related quality of life; MCS, mental component scale; PCS, mental component scale; RR, risk ratio; SD, standard deviation.

[a]The adjusted variables include age, sex, SES, primary kidney disease, and donor type.

[b]The adjusted variables include age, sex, SES, primary kidney disease donor type, and comorbidities.

[c]The total effect is the sum of the direct and indirect effects.
